# Long-Term Administration of Omeprazole-Induced Hypergastrinemia and Changed Glucose Homeostasis and Expression of Metabolism-Related Genes

**DOI:** 10.1155/2024/7747599

**Published:** 2024-05-30

**Authors:** Alina Kabaliei, Vitalina Palchyk, Olga Izmailova, Viktoriya Shynkevych, Oksana Shlykova, Igor Kaidashev

**Affiliations:** Poltava State Medical University, Poltava, Ukraine

## Abstract

**Introduction:**

PPIs, or proton pump inhibitors, are the most widely prescribed drugs. There is a debate regarding the relationship between long-term PPI use and the risk of type 2 diabetes mellitus (T2DM). A potential connection between T2DM and PPIs could be an elevated gastrin concentration. This study is aimed at investigating the long-term effects of PPI omeprazole (OZ) on glucose homeostasis and pancreatic gene expression profile in mice.

**Methods:**

Healthy adult male BALB/c mice were randomly divided into three equal groups (*n* = 10 in each one): (1) experimental mice that received OZ 20 mg/kg; (2) control mice that received 30 *μ*l saline per os; (3) intact mice without any interventions. Mice were treated for 30 weeks. Glucose homeostasis was investigated by fasting blood glucose level, oral glucose tolerance test (GTT), insulin tolerance test (ITT), and basal insulin resistance (HOMA-IR). Serum gastrin and insulin concentration were determined by ELISA. Expressions of *Sirt1*, *Pparg*, *Nfκb1 (p105)*, *Nfe2l2*, *Cxcl5*, *Smad3*, *H2a.z*, and *H3f3b* were measured by RT-PCR.

**Result:**

The ROC analysis revealed an increase in fasting blood glucose levels in OZ-treated mice in comparison with control and intact groups during the 30-week experiment. A slight but statistically significant increase in glucose tolerance and insulin sensitivity was observed in OZ-treated mice within 30 weeks of the experiment. The mice treated with OZ exhibited significant increases in serum insulin and gastrin levels, accompanied by a rise in the HOMA-IR level. These animals had a statistically significant increase in *Sirt1*, *Pparg*, and *Cxcl5* mRNA expression. There were no differences in *β*-cell numbers between groups.

**Conclusion:**

Long-term OZ treatment induced hypergastrin- and hyperinsulinemia and increased expression of *Sirt1*, *Pparg*, and *Cxcl5* in mouse pancreatic tissues accompanied by specific changes in glucose metabolism. The mechanism of omeprazole-induced *Cxcl5* mRNA expression and its association with pancreatic cancer risk should be investigated.

## 1. Introduction

Proton pump inhibitors (PPIs) are the most frequently prescribed medications, commonly used in the treatment of peptic acid disorders [1]. Long-term PPI use has been linked to an increased risk of bone diseases [2], gastrointestinal infections [3], and type 2 diabetes mellitus (T2DM) [4, 5]. Patients with T2DM and concomitant cardiovascular issues frequently used PPIs to stop or prevent gastrointestinal bleeding while undergoing anticoagulant and antiplatelet therapy [6]. Furthermore, the composition of the gut microbiota may influence the relationship between PPI use and T2DM by causing changes in the host's metabolism and gut inflammation [7, 8].

There is a debate regarding the relationship between long-term PPI use and the risk of T2DM. Long-term PPI use was linked to a 24% higher risk of diabetes, according to a prospective analysis [5]. According to a prospective population-based cohort study, using PPIs increased the risk of T2DM by 1.7 times [4]. A different nested case-control study found that patients with upper gastrointestinal illness who took PPIs at higher doses had a dose-dependently higher risk of developing T2DM [9].

However, other studies have found the opposite, showing no link between PPIs and T2DM [10, 11]. Other studies have demonstrated the safety and efficacy of PPIs in the treatment of acid peptic disease in patients with T2DM [12, 13].

In nonobese diabetic mice, the combination of GABA, sitagliptin, and omeprazole (OZ) prevented the onset of type 1 diabetes and promoted its reversal [14]. A plausible association between PPIs and diabetes mellitus could be attributed to elevated levels of gastrin, which stimulates the regeneration of pancreatic *β*-cells, in both pancreatic tissue and blood as a result of H^+^/K^+^-ATPase inhibition [15–17].

OZ remains the most used PPI in almost all countries [18] as well as the most widely used PPI in animals [19]. Surprisingly, there is limited data about the influence of OZ treatment on pancreatic gene expression profiles limited by regenerating islet-derived-3 gamma (Reg3*γ*), leucine-rich *α*2 glycoprotein (Lrg), regenerating islet-derived-3 alpha (Reg3*α*), pancreatitis-associated protein (Pap), Muclin, and IL-13 receptor-alpha genes [20]. On the other hand, there is data about the involvement of other metabolism, inflammation [21–26], and histone [27] genes in the pathogenesis of T2DM.

Thus, this study is aimed at exploring the long-term effects of OZ on glucose homeostasis and metabolism- and inflammation-related and histone pancreatic gene expression profiles in mice.

## 2. Materials and Methods

### 2.1. Animals and Study Design

The Ethics Committee of Poltava State Medical University, Poltava, Ukraine, approved the animal studies (Minutes No. 207 as of 23.08.2022).

Standard cages were utilized to house healthy adult male BALB/c mice weighing 21 ± 2 g. The temperature was kept at room temperature (19 ± 2°C) with a relative humidity of 63 ± 6% and a light/dark cycle of 12/12 hours. Water and food were available to them at all times. The research was made available from September through April.

Three equal groups of ten mice each were randomly assigned: group 1 was for experimental mice, which received OZ 20 mg/kg diluted in 30 *μ*l saline per os; group 2 was for control mice, which received 30 *μ*l saline per os; group 3 was for intact mice, which received no interventions. The animals received treatment for a duration of thirty weeks.

Mice were fasted overnight, and on day 210, thiopental sodium 50 mg/kg intraperitoneally was used to induce anesthesia. Samples of heart blood were taken, and the sera were kept at -20°C. Samples of the pancreas were obtained and kept at -70°C.

### 2.2. Assessing Glucose Homeostasis in Mice

The fasting blood glucose levels were measured once a week (0, 5, 9, 14, 18, 24, 26, and 28 weeks). The oral glucose tolerance test (GTT) was performed after oral glucose intake (2 g/kg) as described [28] on days 126 and 183. The insulin tolerance test (ITT) was performed by the intraperitoneal injection of insulin 0.5 U/kg (Gensulin 100 U/ml, BIOTON, Poland) [29] on days 169 and 198. The measurement of fasting blood glucose level and GTT/ITT was performed on different days (with 2-day intervals) during one experimental week. Mice underwent tail-pinch acclimation training one week before the first blood glucose measurement. Glucose concentration was measured in blood samples obtained from the tip of the tail by a hand-held glucometer (Accu-Chek Active, Germany, Art No. 04454642001).

For estimation of the homeostasis model assessment of basal insulin resistance (HOMA-IR), the following equation was used [28]:

(1) $$\begin{array}{c} \text{HOMA}‐\text{IR}=\frac{\text{fasting insulin level }\left(\mu \text{U}/\text{ml}\right)\times \text{fasting blood glucose }\left(\text{mmol}/\text{l}\right)}{22.5}. \end{array}$$

### 2.3. Serum Hormone Assay

Serum gastrin concentrations were measured after overnight fasting by Gastrin EIA Kit (Sigma-Aldrich, USA; Cat. No. RAB0200), and insulin—Mouse Insulin ELISA Kit (Thermo Fisher, USA; Cat. No. EMINS). All measurements were provided with LabLine-026 (Labline, Austria) Photometer.

### 2.4. RNA Preparation and Quantitative Reverse Transcription PCR

For RNA extraction, pancreatic tissue samples were collected from all mice at each timepoint. The RNeasy kit (QIAGEN, Germany), Cat. No. 74104, was used to extract total RNA from the pancreatic tissue.

We generated single-strand DNAs by reverse transcribing approximately 1 *μ*g of total RNA from each sample using the QuantiTect® Reverse Transcription Kit (QIAGEN, Germany), Cat. No. 205313.

For SYBR Green-based analysis, the QuantiTect® SYBR-Green PCR Kit (QIAGEN, Germany), Cat. No. 204143, was used to amplify the cDNA equivalent of 50 ng of total RNA from each sample in the CFX96TM RealTime PCR Detection System (Bio-Rad, USA).

Data accuracy was ensured by analyzing each sample in duplicate. The gene expressions were detected as 2^−ΔCt^, and all values were normalized to the expression of the housekeeping gene *Gadph* and *Sirt1* that was normalized to *Rpl19* as described [30].

Table 1 displays the precise primer sequences used in the real-time PCR. The source of all the oligonucleotides was Metabion International AG in Germany.

All pairs of primers were checked for specificity by Primer-BLAST software (https://www.ncbi.nlm.nih.gov/tools/primer-blast/index.cgi). Specificity of primers was estimated as follows: (1) at least one primer (for a given primer pair) must have two or more mismatches to unintended targets in the last five bases at the 3′ end, and (2) any targets with six mismatches or more to at least one primer (for a given primer pair) should be ignored [37]. Primers' pairs for *Sirt1*, *Smad3*, *H2a.z*, and *Nfe2l2* had up to 5 mismatches between at least one of the primers and the targets and were additionally scrutinized by amplicon size using automatic capillary electrophoresis system QIAxcel Advanced (QIAGEN, Germany).

### 2.5. Histological Study

Pancreatic samples were taken in all animals. Tissues were fixed in Bouin's solution for 15 min and embedded in paraffin blocks. 5 *μ*m sections were stained by Mallory trichrome staining. Histological parameters, including the diameters of islets, the number of *β*-cells, the tonality of insulin granules within the cytoplasm of *β*-cells, and cytoplasmic vacuolization, were evaluated.

In each slide, islets were divided into two categories (based on median): small islets (<80 *μ*m in diameter) with little space and fewer *β*-cells per islet and large islets (>80 *μ*m in diameter) with open spaces and a high number of *β*-cells [38–40].

For *β*-cells counting, 30 slides from the pancreas of each group were randomly selected. Then, 10 microscopic fields of equal size were screened. The number of *β*-cells in large and small islets, separately, was assessed by counting all nuclei of blue-stained cells inside one islet in the field [41].

All measurements were performed under a light microscope using Axio Lab.A1 (Carl Zeiss, Göttingen, Germany) and Zen 2.5 lite (blue edition) software.

### 2.6. Statistical Analysis

Data analyses were done using GraphPad Prism 5.0 software (San Diego, USA). The data are reported as mean ± standard deviation (SD) or median (M) (25-75%). One-way ANOVA followed by Tukey's or Dunn's posttests for multiple comparison were used to assess the variations in means among the groups. The receiver operating characteristic (ROC) curve and the area under the ROC curve (AUC) were used for evaluating the difference in body weight, fasting blood glucose level, and GTT/ITT experiments. To assess the degree of relationship between the concentrations of insulin and gastrin in blood serum, the cluster analysis was provided. MacQueen's k-means clustering algorithm was used, utilizing Manhattan distance estimation. The level of significance for all tests was set at *P* < 0.05.

## 3. Results

### 3.1. Influence of OZ on Body Weight and Glucose Homeostasis in Male Mice

**Figure 1:**
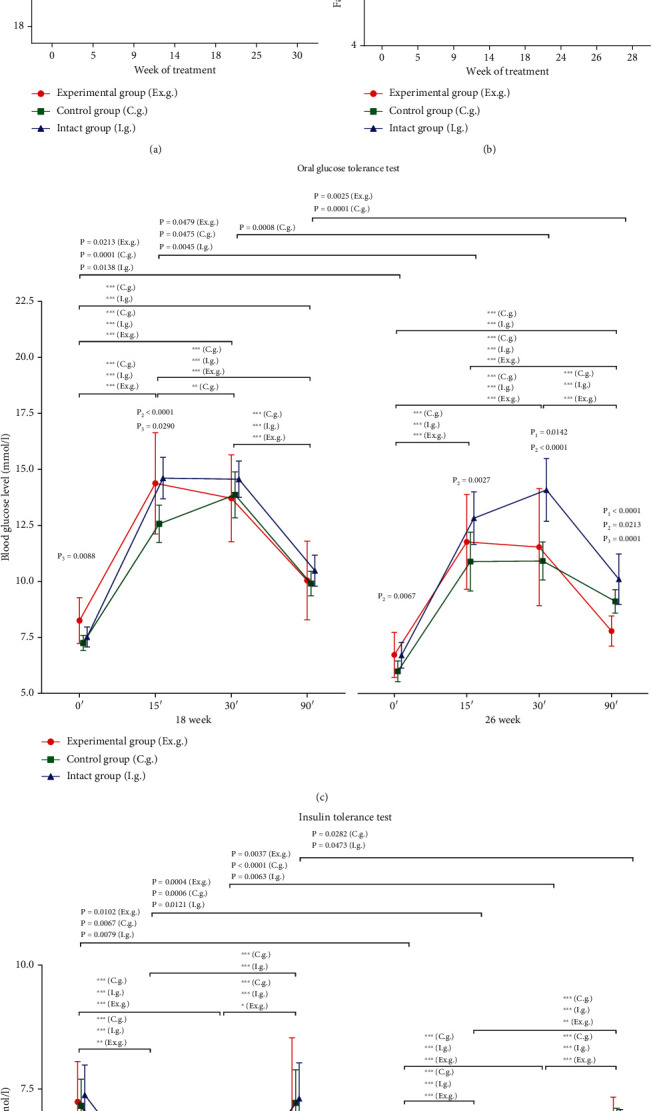
Effects of OZ treatment on (a) body weight, (b) serum glucose levels, (c) oral glucose, (d) and insulin tolerance tests in male BALB/c mice. Comparison of data in the group at different time points: ^∗^*P* < 0.05; ^∗∗^*P* < 0.01; ^∗∗∗^*P* < 0.001. *P*_1_: comparison of experimental and intact groups; *P*_2_: comparison of control and intact groups; *P*_3_: comparison of experimental and control groups.

In intact mice, we observed a permanent significant increase in body weight during 30 weeks of the observation (Figure 1(a)). Similar dynamics of increasing body weight were registered in control mice as well as in mice treated with OZ. In the experimental group, body weight was significantly increased at 9 weeks of the observation; but at further time points, this increase disappeared. During the whole experiment, there were no statistically significant differences in body weight AUC between experimental and control or intact groups (AUC 0.5703 (95% CI: 0.4724-0.6682; *P* = 0.1511) and AUC 0.5282 (95% CI: 0.4311-0.6252; *P* = 0.5652), respectively).

The fasting blood glucose levels had no statistically significant differences at week 0 among the three groups. The fasting blood glucose levels were increased from 9 until 28 weeks of the observation in all investigated groups of mice (Figure 1(b)). The elevations observed were notably more pronounced in the experimental group compared to control and intact mice. To estimate intergroup differences in the fasting blood glucose levels during the whole observation period, the ROC analysis was provided. We observed a statistically significant increase in the fasting blood glucose levels in the experimental group in comparison with control and intact groups (AUC 0.6395 (95% CI: 0.5536-0.7253, *P* = 0.0023) and AUC 0.7286 (95% CI: 0.6500-0.8072, *P* = 0.0001), respectively).

After oral glucose administration, the normal reactivity patterns with increasing levels of blood glucose levels at 15 and 30 minutes (Figure 1(c)) were observed in all groups of mice. There was a statistically significant increase in blood glucose level in the experimental group in comparison with the control group at 15 minutes. Similar results of the oral glucose test were observed at 26 weeks of the experiment. We did not observe statistically significant differences in GTT between control and experimental groups (AUC 0.5622 (95% CI: 0.4352-0.6891); *P* = 0.3384), control and intact groups (AUC 0.6113 (95% CI: 0.4863-0.7362); *P* = 0.08681), and intact and experimental groups (AUC 0.5250 (95% CI: 0.3961-0.6539); *P* = 0.7003) at the 18th week. In contrast, there were statistically significant differences in GTT between control and intact groups (AUC 0.6806 (95% CI: 0.5610-0.8002); *P* = 0.005) and intact and experimental groups (AUC 0.6309 (95% CI: 0.5074-07545); *P* = 0.044) at the 26th week.

After insulin injection in experimental mice, there was a statistically significant decrease in blood glucose concentrations at 15 and 30 minutes at all time points (24 and 28 weeks) (Figure 1(d)). We observed a slight increase in insulin sensitivity in the experimental group in comparison with the intact group at 28 weeks. There were no statistically significant intergroup differences in ITT levels at the 24th week of the experiment. At the 28th week, we observed statistically significant differences in ITT results between control and experimental groups (AUC 0.6147 (95% CI: 0.4894-0.7400); *P* = 0.0475) and intact and experimental groups (AUC 0.6544 (95% CI: 0.5301-0.7787); *P* = 0.0175).

### 3.2. Influence of OZ on Basal Insulin Resistance, Serum Gastrin, and Insulin Levels in Male Mice

**Figure 2:**
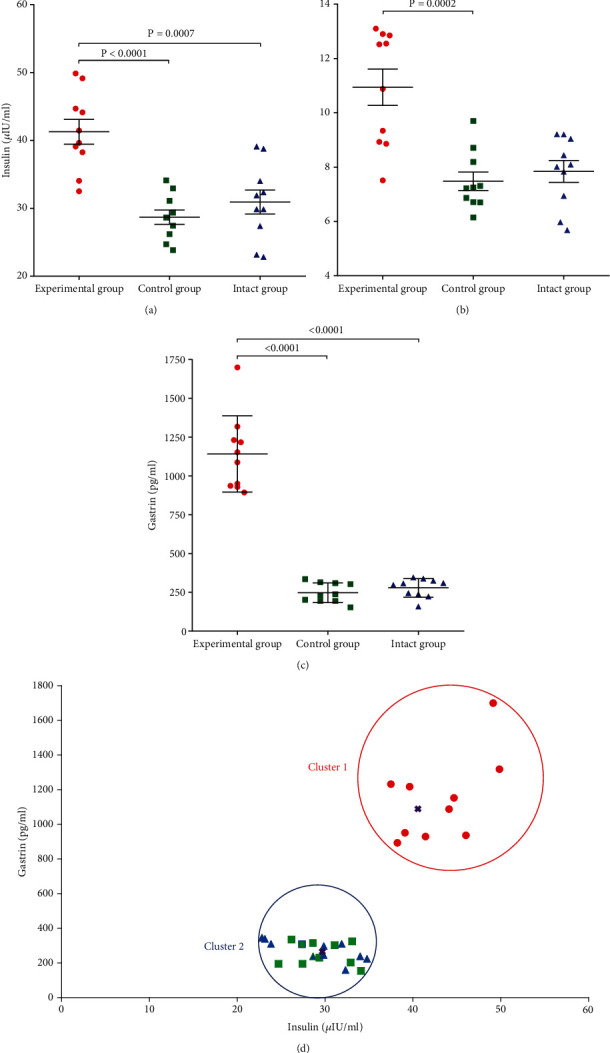
Effects of OZ treatment on (a) basal insulin resistance (HOMA-IR), (b) serum gastrin, (c) and insulin levels in male BALB/c mice and (d) cluster analysis of serum insulin and gastrin concentrations in experimental, control, and intact groups.

After 30 weeks of OZ administration, we observed a statistically significant increase in serum insulin concentration in comparison with control and intact groups (Figure 2(a)). At the same time, male mice treated with OZ had significantly higher levels of HOMA-IR in comparison with other groups (Figure 2(b)). The serum gastrin concentration in mice treated with OZ was significantly elevated in comparison with control and intact groups (Figure 2(c)).

We conducted a cluster analysis to examine the relationship between serum insulin and gastrin levels. The provided analysis revealed two clusters: cluster 1 comprised 10 experimental animals treated with OZ, and cluster 2 comprised 20 control and intact animals. Cluster 1 had cluster centers 41.276 (insulin) and 1141.25 (gastrin), and cluster 2 had 29.8045 (insulin) and 262.955 (gastrin). The ANOVA was implemented to compare two clusters to see for which variables the clusters are significantly different from one another. Clusters 1 and 2 exhibited differences in both insulin and gastrin variables (*P* = 0.01) (Figure 2(d)).

### 3.3. Influence of OZ on the mRNA Expression of Metabolism/Inflammation-Related and Chromatin-Modifying Genes

**Figure 3:**
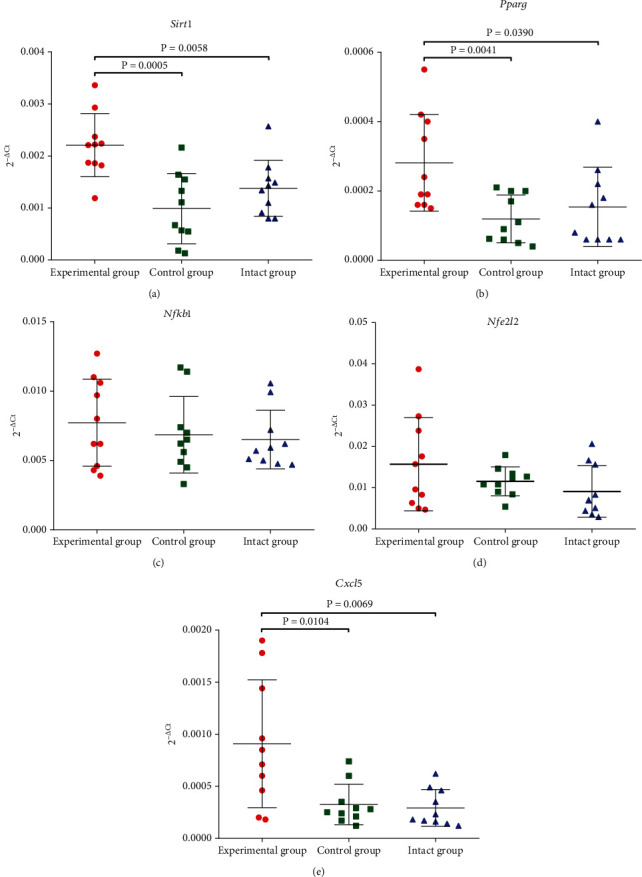
Effects of OZ treatment on the mRNA expression of inflammation-related genes: (a) *Sirt1*, (b) *Pparg*, (c) *Nfκb1*, (d) *Nfe2l2*, (e) and *Cxcl5* in the mouse pancreas.

After 30 weeks of OZ administration, we observed a statistically significant increase in *Sirt1*, *Pparg*, and *Cxcl5* expression in comparison with the control and intact group. Administration of OZ did not influence *Nfκb1* and *Nfe2l2* expression (Figure 3).

**Figure 4:**
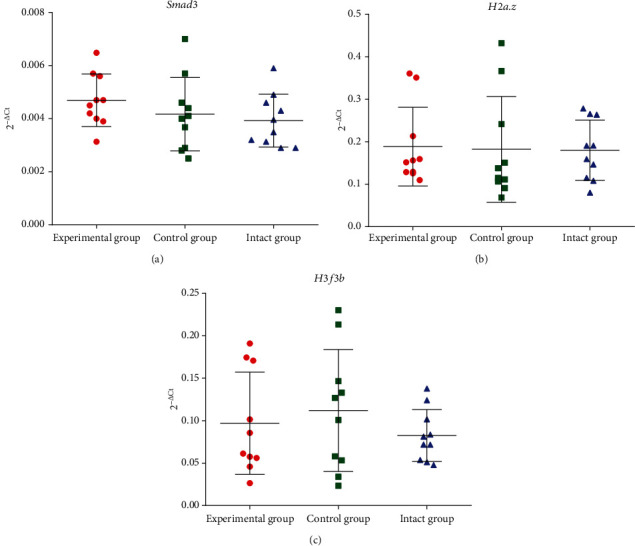
Effects of OZ treatment on the mRNA expression of chromatin-modifying genes: (a) *Smad3*, (b) *H2a.z*, (c) *H3f3b*.

Figure 4 shows the expression pattern of chromatin-modifying genes in mouse pancreas. There were no differences in *Smad3*, *H2a.z*, and *H3f3b* expression after OZ treatment in the mouse pancreas (Figure 4).

### 3.4. Effects of OZ Treatment on Mouse Pancreas Histology

Islets can vary in size, and hyperplasia is common in aging mice [42]. It has been suggested that islets may exhibit functional differences related to their size [38]. In response to increased demand for insulin, pancreatic *β*-cells may adapt by augmenting insulin secretion, either through increased *β*-cell function, increased *β*-cell mass, or both. For example, it is well established that the leptin-deficient ob/ob mouse exhibits both of these features [38]. However, it remains unclear if islets of different sizes can change their *β*-cell numbers in conditions of hypergastrinemia.

**Figure 5:**
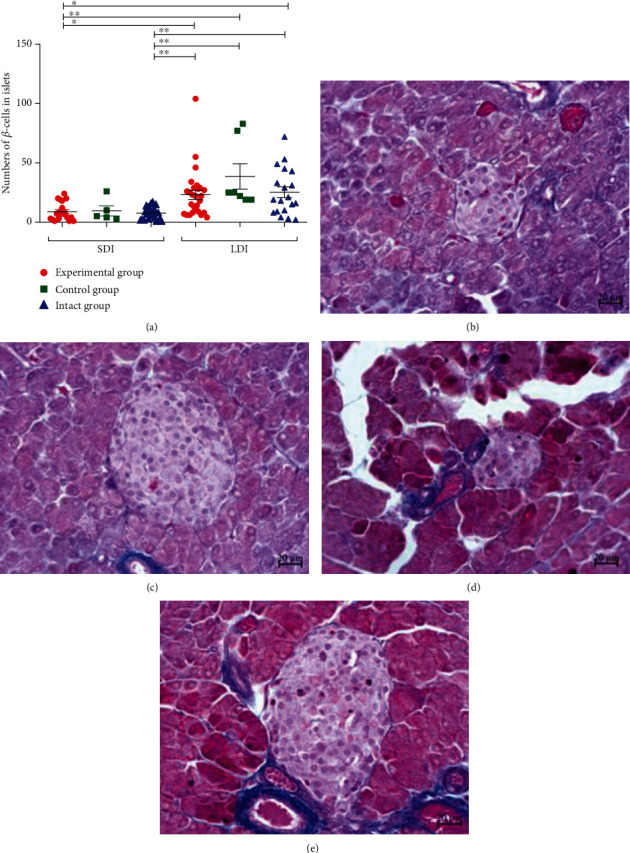
Comparison of *β*-cell counts between groups depending on islet diameters: (a) one-way ANOVA (Kruskal-Wallis statistic) and posthoc Dunn's multiple comparison test, (b, c) representative islets from experimental mouse, and (d, e) representative islets from intact mouse.

In the experimental group, *β*-cell numbers in small-diameter islets (SDI) were significantly lower compared to large-diameter islets (LDI). Additionally, *β*-cell numbers in SDI were significantly lower compared to LDI of two other groups (Figure 5). In the control group, significant differences were not revealed in this study. In the intact group, *β*-cell numbers in SDI were significantly lower compared to LDI of the experimental, control, and intact groups. The study identified between-group differences, as depicted in Figure 5. The numbers of *β*-cells in the islets were directly related to the size of the islets in the experimental and intact groups.

## 4. Discussion

The primary mechanism by which OZ influences glucose metabolism involves elevating the endogenous gastrin level, which promotes *β*-cell regeneration and enhances glucose tolerance [43]. OZ has been shown to function as an adjuvant with hypoglycemic medications and, as previously reported, improved glycemic control by raising pancreatic and blood levels of gastrin [44–46]. By blocking the *β*-cell insulin granules' V-ATPase proton pump, OZ enhanced insulin secretion [47]. In our study, following a 30-week course of OZ treatment, we found statistically significant increases in the serum levels of both insulin and gastrin. The increased serum secretions of insulin and gastrin were not accompanied by the morphological changes of islet structure and *β*-cell number in the mouse pancreatic tissues.

Gastrin has pleiotropic effects on glucose homeostasis and pancreatic physiology. Both healthy and malignant gastrointestinal tissues are subject to the growth-promoting effects of this hormone [48–51]. The idea that gastrin stimulates islet growth only when other growth factors are present is supported by transgenic studies [52]. On the other hand, by stimulating glucagon secretion, gastrin may support glucose homeostasis in adults. The physiological release of glucagon in response to gastrin has been associated with the expression of CCK2 receptors on adult pancreatic glucagon cells [53, 54]. We observed an increase in the fasting blood glucose levels at 30 weeks after OZ treatment, as well as a decrease in glucose tolerance, and a slight increase in insulin sensitivity. Notably, there was an increase in HOMA-IR by the 30th week of OZ treatment. These findings suggest that omeprazole-induced hypergastrinemia exerted a complicated influence on glucose homeostasis by the induction of insulin secretion. Literature data and our results suppose the possible role of glucagon in the influence of OZ-induced hypergastrinemia on glucose homeostasis. Although glucagon functions as a counter-regulator to insulin and is the second major hormone involved in maintaining glucose homeostasis, research exploring the impact of nutrients and external factors on glucagon secretion, biosynthesis, and gene expression has been relatively limited compared to studies on insulin. On the other hand, T2DM patients frequently have chronic hyperglucagonemia, which contributes to hyperglycemia [55]. The disbalance in gastrin/insulin synthesis was displayed by the cluster analysis after 30-week OZ treatment in mice, as shown in the current experiment. OZ-treated mice had statistically significant higher serum gastrin and insulin levels.

Hypergastrin- and hyperinsulinemia together with glucose homeostasis changes had a powerful pleiotropic influence on metabolism- and inflammation-related genes. Our study observed a statistically significant increase in mRNA levels of *Sirt1*, *Pparg*, and *Cxcl5* in the mouse pancreatic tissues after OZ treatment.

By deacetylating a group of proteins involved in inflammatory response, longevity, and metabolic homeostasis, SIRT1 promotes the expression of genes resistant to stress and protects against injury [56–58]. It was discovered that SIRT1 can upregulate GLUT1 expression [59] and is a negative regulator of glycolysis in the liver and skeletal muscles [60]. SIRT1 shields *β*-cells against nitric oxide damage [61] and cytokine-mediated apoptosis [62, 63]. By directly suppressing the *Ucp2* gene, *Sirt1* may operate as a positive regulator of insulin secretion in response to glucose [64]. *Sirt1* controls the expression of certain mitochondria-related genes that govern metabolic coupling in *β*-cells; a reduction in *Sirt1* expression in *β*-cells compromises insulin secretion and glucose sensing [65]. Increased insulin secretion and improved glucose tolerance were observed in a mouse model where *Sirt1* was overexpressed specifically in *β*-cells [66]. A growing body of research suggests that SIRT1 protects against lipotoxicity and glucotoxicity [67]. Our experimental data showed that OZ treatment might induce *Sirt1* mRNA expression in the mouse pancreas.

SIRT1 can interact with peroxisome proliferator-activate receptor gamma (PPARG), a protein related to metabolism. SIRT1 also represses PPARG, which is a crucial regulatory protein [68]. By attaching itself to the nuclear receptor corepressor and silencing the mediator of the retinoid and thyroid hormone receptor, SIRT1 represses PPARG [69]. Peroxisome-proliferation activator receptor gamma coactivator 1 alpha (PGC1A) is a negative regulator of glycolysis that is stimulated by SIRT1 deacetylation [70]. Concurrently, increased PGC1A expression in islets suppressed important genes related to glucose metabolism, resulting in a significant decrease in insulin secretion [71]. In our study, we did not observe such inhibition of *Pparg* mRNA expression by enhanced expression of *Sirt1* mRNA. Our observation goes in parallel with data that gastrin can upregulate *Pparg* gene expression [72, 73].

Taking together, these findings suppose that long-lasting omeprazole-induced hypergastrinemia leads to the specific functional status of glucose homeostasis, characterized by hyperinsulinemia with slight insulin tolerance, as well as impaired interaction between SIRT1 deacetylase and transcriptional factor *Pparg* in the mouse pancreas.

The chemokine CXCL5 was chosen as a hub gene because of its strong correlations with CTLA4, PDCD1, PDCD1LG2, CD274, and PDCD1. In addition to possibly playing a role in oncogenesis, tumor invasion, and metastasis in a range of pancreatic malignancies, CXCL5 can affect apoptosis in islet cells [74]. Among the CXCR2 ligands, CXCL5 demonstrated the highest expression levels in human pancreatic ductal adenocarcinoma. The expression of *Cxcl5* was linked to the expression of mutant *Kras* and controlled by activation of NF*κ*B [75]. For patients with pancreatic cancer, *Cxcl5* expression may be a reliable prognostic biomarker [76]. By triggering the Jak2/STAT5/SOCS2 pathway, *Cxcl5* inhibits insulin signaling [77] and causes insulin resistance in a variety of tissues [78, 79]. The existing literature does not provide conclusive evidence regarding the potential impact of glucagon and gastrin on the expression of *Cxcl5* in islets. On the other hand, some diabetes medications, like the dipeptidyl peptidase-4 inhibitor sitagliptin, suppress CXCL5 and do not hasten the development of intestinal neoplasia in mice [80]. This data is consistent with reports [81–84] suggesting a possible link between pancreatic cancer risk and long-term proton pump inhibitor therapy. In contrast, by specifically targeting H^+^/K^+^-ATPases in pancreatic cancer cells, PPIs may slow the progression of pancreatic adenocarcinoma [85, 86]. Our finding that long-lasting OZ treatment might enhance *Cxcl5* mRNA expression supposes the possible importance of this mechanism for the omeprazole-related pancreatic cancer risk.

We hypothesized that such complicated interactions might involve other transcriptional factors and networks. Expression of *Smad3*, *H2a.z*, and *H3f3b* mRNA was investigated. Diabetes may cause dysregulation of *Smad3* and *Tgfb*, two crucial regulators of insulin gene transcription and *β*-cell function [87]. It has been shown that the histone variants H2a.z and H3.3, which are both necessary for multicellular organisms, have important and distinct functions in controlling chromatin structure and function during development and in diseases [88–90]. Insulin-induced acetylation of H2a.z and H3k9/14 at the promoter of hexokinase 2 (HK2) doubled the amount of the enzyme's expression [91]. Additionally, H2a.z contributes to the oncogenic 3D genome organization and the biology and development of pancreatic cancer by generating a prooncogenic transcriptome through posttranslational modifications, interactions with various partners, and regulatory elements [92]. In both normal and pancreatitis conditions, the regulation of pancreatic cell genome activity is influenced by histone variant *H3f3b* [93]. This histone participated in the realization of gastrin effects [94], as well as the regulation of gene expression in individuals with metabolic syndrome [94, 95]. In our study, 30-week OZ treatment did not induce changes in mRNA expressions of *Smad3*, *H2a.z*, and *H3f3b* genes.

Our study encountered several limitations, notably the absence of an additional experimental group with OZ treatment in mice with impaired glucose metabolism, as well as the lack of data concerning protein expressions and their functional activities. The glucagon level was not assessed, and other organs (liver and muscles) might be a target for OZ activity and interfere with glucose homeostasis. Furthermore, islet morphology analysis was limited by light microscopy.

Further investigations are needed to elucidate the complicated mechanism of OZ's influence on glucose metabolism with respect to hypergastrin- and hyperinsulinemia in normal mice and mice with impaired carbohydrate metabolism. Similar studies should be conducted to evaluate the long-term impact of OZ treatment on patients with different clinical conditions (metabolic syndrome, systemic inflammation, prediabetes, etc.). We need additional investigations of H^+^/K^+^-ATPase inhibition and *β*-cell physiology after long-term OZ treatment.

## 5. Conclusions

In conclusion, long-term OZ treatment induced hypergastrin- and hyperinsulinemia increased expression of *Sirt1*, *Pparg*, and *Cxcl5* mRNA accompanied by specific changes in glucose metabolism. Mechanisms of omeprazole-induced *Cxcl5* mRNA expression and its association with pancreatic cancer risk should be investigated.

## Figures and Tables

**Table 1:** Primer sequences for mRNA measurement.

Gene	Primer sequences	Oligo ID	References
*Sirt1*	Forward TCTGTCTCCTGTGGGATTCC Reverse GATGCTGTTGCAAAGGAACC	211116B056C04 27/87 211116B056D04 28/87	[30]
*Pparg*	Forward CCAGAGCATGGTGCCTTCGCT Reverse CAGCAACCATTGGGTCAGCTC	211116B056G04 31/87 211116B056H04 32/87	[31]
*Nfκb1 (p105)*	Forward GAGGTCTCTGGGGGTACCAT Reverse AAGGCTGCCTGGATCACTTC	211116B056A05 33/87 211116B056B05 34/87	
*Nfe2l2*	Forward СGCCGCCTCACCTCTGCTGCCAGTAG Reverse AGCTCATAATCCTTCTGTCG	211116B056C05 35/87 211116B056D05 36/87	[32]
*Cxcl5*	Forward TGCCCTACGGTGGAAGTCAT Reverse AGCTTTCTTTTTGTCACTGCCC	211116B056E05 37/87 211116B056F05 38/87	[33]
*Smad3*	Forward GCTGGGCATGCTGATCCT Reverse CCCATGGTCTGTGCCCTTT	211116B056G05 39/87 211116B056H05 40/87	[34]
*H2a.z*	Forward TATCACCCCTCGTCACTTGC Reverse TCCACTGGAATCACCAACAC	211116B056A06 41/87 211116B056B06 42/87	[35]
*H3f3b*	Forward CTGAGAGAGATCCGTCGTTACC Reverse CTTCAACTTAAGCTCTCTCCC	211116B056C06 43/87 211116B056D06 44/87	
*Rpl19*	Forward AAGCCTGTGACTGTCCATTC Reverse CTTCTTGGATTCCCGGTATC	211116B056E04 29/87 211116B056F04 30/87	[30]
*Gadph*	Forward GCACAGTCAAGGCCGAGAAT Reverse GCCTTCTCCATGGTGGTGAA	211116B056C02 11/87 211116B056F02 14/87	[36]

## Data Availability

The datasets generated during and/or analyzed during the current study are available from the corresponding author upon reasonable request.
